# ‘Elevated’ hemidiaphragm due to a pericardial cyst

**DOI:** 10.1007/s12471-015-0792-4

**Published:** 2015-12-21

**Authors:** V.A.P. Borghouts, Y.J. Stevenhagen, L.J. Wagenaar, D.E. Bouman, P.M.J. Verhorst

**Affiliations:** 1 Department of Cardiology, Thoraxcentrum Twente, Medisch Spectrum Twente, Enschede, The Netherlands; 2 Department of Radiology, Medisch Spectrum Twente, Enschede, The Netherlands

A 60-year-old patient was seen in our gastroenterology outpatient clinic for the evaluation of abnormal liver function tests. Chest X-ray demonstrated what was interpreted as an elevated right hemidiaphragm (Fig. 1). Abdominal sonography revealed a fluid collection above the liver.

**Fig. 1:**
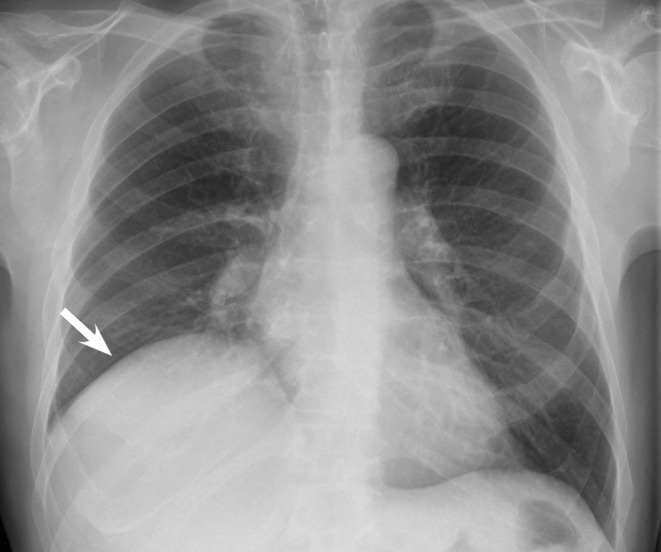
Upright chest x-ray demonstrating what seemed to be an elevated right hemidiaphragm (*arrow*).

A contrast-enhanced computed tomography (CT) scan of chest and abdomen was performed and revealed a non-enhancing, fluid density large supradiaphragmatic mass located in the right cardiophrenic angle, with close relation to the pericardium. Cardiac magnetic resonance imaging (CMR) of the thoracic cavity confirmed the mass to be a 17 × 13 cm pericardial cyst (Fig. 2).

**Fig. 2:**
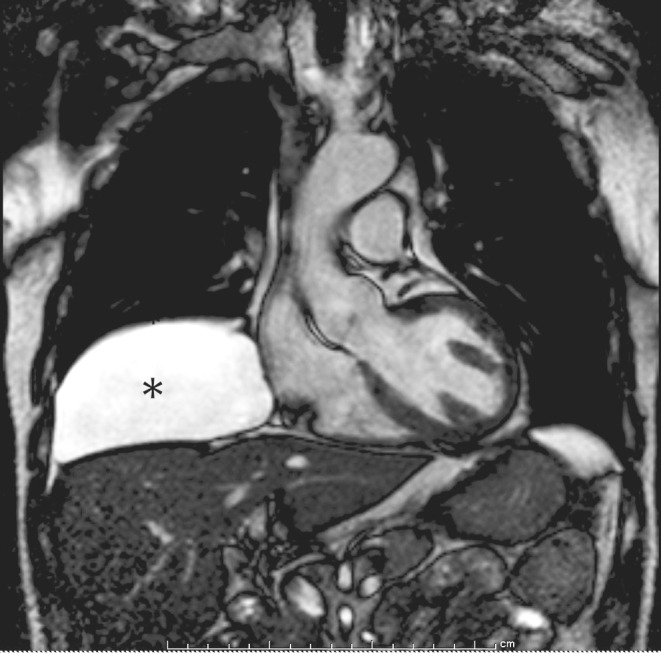
Cardiac magnetic resonance imaging showing a large mass outside the pericardium, with high signal intensity on T2-weighed imaging, most consistent with a pericardial cyst (*asterisk*).

## Discussion

With an incidence of approximately 1 in 100,000, pericardial cysts are rare mediastinal masses [1]. Seventy percent of pericardial cysts are located at the right cardiophrenic angle [1, 2]. Most patients are asymptomatic [1, 3], resulting in the diagnosis of a pericardial cyst as an incidental finding on thoracic imaging studies [1–3]. Compression of the heart can cause symptoms resulting in chest discomfort, dyspnoea or palpitations [1, 3–5]. In symptomatic patients, percutaneous aspiration and ethanol sclerosis can be suggested, as well as surgical resection [3, 5]. In asymptomatic patients, no treatment is necessary [5]. Annual observation is optional and can be performed by echocardiography, CT or CMR.

### Funding

None.

### Conflict of interest

None declared.

## References

[1] Patel J, Park C, Michaels J, Rosen S, Kort S (2004). Pericardial cyst: case reports and a literature review. Echocardiography.

[2] Yared K, Baggish AL, Picard MH, Hoffmann U, Hung J (2010). Multimodality imaging of pericardial diseases. JACC Cardiovasc Imaging.

[3] Bezgin T, Elveran A, Varol S, Doğan C, Karagöz A, Esen AM (2014). Pericardial cyst. Herz.

[4] Kumar Paswan A, Prakash S, Dubey RK (2014). Cardiac tamponade by hydatid pericardial cyst: a rare case report.. Anesth Pain Med.

[5] Nayak K, Shetty RK, Vivek G, Pai UM. Pericardial cyst: a benign anomaly. BMJ Case Rep. 2012. doi:10.1136/bcr-03-2012-5984. 10.1136/bcr-03-2012-5984 PMC4543131 22949119

